# Individualizing the choice of surgical therapy for gastroesophageal reflux disease

**DOI:** 10.1097/MOG.0000000000001082

**Published:** 2025-02-17

**Authors:** Luigi Bonavina

**Affiliations:** IRCCS Policlinico San Donato, Division of General and Foregut Surgery, Department of Biomedical Sciences for Health, University of Milan, Milano, Italy

**Keywords:** antireflux surgery, Barrett's esophagus, crural repair, esophago-gastric junction, fundoplication, gastroesophageal reflux disease, hiatus hernia

## Abstract

**Purpose of review:**

Proton-pump inhibitor therapy does not provide complete relief of symptoms in up to 40% of patients with gastroesophageal reflux disease (GERD). Antireflux surgery (ARS) aims to reconstruct the natural antireflux barrier consisting of the diaphragmatic crura, the lower esophageal sphincter, and the gastroesophageal flap valve.

**Recent findings:**

Although the 360° Nissen fundoplication combined with crural repair remains the gold-standard ARS treatment for GERD, the Toupet and Dor partial fundoplications and the magnetic sphincter augmentation (LINX) procedure have emerged as suitable alternative options with fewer side-effects. Randomized and observational clinical studies show that reflux control with partial fundoplications and LINX is acceptable and the risk of side-effects is minimal. Early results with the novel Refluxstop procedure show that restoration of distal esophageal length and the gastroesophageal flap valve, combined with anterior fundoplication and a silicon prosthesis to stabilize the esophagogastric junction below the diaphragm, can also provide excellent reflux control with minimal side-effects.

**Summary:**

Laparoscopic ARS should be performed in centers offering a comprehensive diagnostic pathway and a spectrum of techniques tailored to the individual GERD patient's phenotype and expectations. Further research is needed to provide more personalized and durable ARS.

## INTRODUCTION

The modern era of antireflux surgery (ARS) started in the early 1950s with the seminal work of Philip Allison and Rudolf Nissen and their contribution toward understanding the role of crural diaphragm and esophago-gastric junction (EGJ) in the pathogenesis of gastroesophageal reflux disease (GERD) [1]. As surgical techniques for treating GERD advanced, the lower esophageal sphincter (LES) soon was recognized as the major barrier against gastroesophageal reflux in humans [2]. Therefore, the Nissen 360° fundoplication became the gold-standard surgical procedure for GERD treatment. In the early 1960s, partial posterior (Toupet) and anterior (Dor) fundoplications were proposed to reduce the risk of postoperative dysphagia seen with the Nissen. In the early 1990s, with the advent of laparoscopy, ARS became minimally invasive and a more attractive therapeutic option for GERD. Unfortunately, high failure rates in the early stages of laparoscopic surgery, coupled with the concurrent introduction of proton pump inhibitors (PPI), led to a steady decline in the utilization of ARS [3]. This occurred despite the high rate of patient dissatisfaction with PPIs, the increasing safety concerns regarding long-term PPI use, and the rising prevalence of GERD in the younger population [4–5]. Presently, GERD remains a chronic debilitating disease with a substantial burden of persistent/refractory symptoms in at least 40% of patients treated with PPIs, and a negative impact on health-related quality of life and healthcare resource utilization [6]. Last, but not least, reflux-induced intestinal metaplasia and dysplasia develop in about 10% of these patients [7] and have the potential to progress to Barrett's adenocarcinoma despite symptomatic control with PPIs. Surprisingly, laparoscopic ARS is offered only to a minority of patients with GERD and has not been fully embraced by the medical community or the public, in part as a result of bad publicity and fear of adverse outcomes [8]. The concept of personalized therapy for GERD emerged in the 1990 s considering the burden of the disease and the dissatisfaction with current treatment modalities. 

**Box 1:**
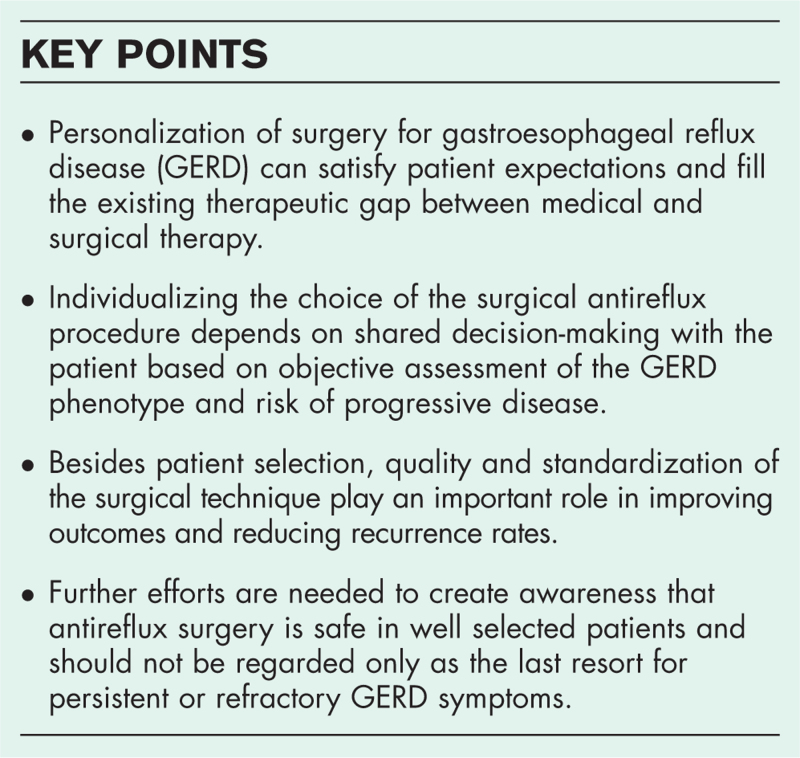
no caption available

## PERSONALIZING GERD SURGERY

### Current understanding of the antireflux barrier

The ideal surgical antireflux technique should provide relief of symptoms and complications of GERD, eliminate or reduce PPI dependency, improve quality of life, and avoid side-effects such dysphagia, bloating, and the inability to belch and vomit. Traditionally, these goals can be achieved by reducing the hiatal hernia, restoring the intra-abdominal esophagus, repairing the crural defect, and augmenting the ‘physiological’ antireflux barrier through a fundoplication. In addition, current evidence supports the antireflux effect of the gastroesophageal flap valve, an anatomical mucosal-muscular fold visible on retroflexed endoscopic view [9]. The American Foregut Society endoscopic classification has recently included the axial hiatal hernia length, hiatal aperture diameter, and presence or absence of the gastroesophageal flap valve as the main anatomical determinants of EGJ competency to be reported at endoscopy [10]. This information is critical for the surgeon, can influence patient selection and tailoring of the antireflux procedure, and can be used to understand patterns of failure [11^▪^].

### Importance of gastroesophageal reflux disease phenotype

Despite the heterogeneous clinical and pathophysiological presentations, GERD patients have historically been treated in a similar way (mostly with PPIs), and the choice of the antireflux procedure has been based on surgeon's preference (typically a Nissen fundoplication). Indication for surgery has generally been reserved to individuals with refractory GERD and no or partial response to PPI. This equivocal approach has led to the existing gap between medical and surgical therapy, indicating the need to optimize the GERD management pathway and to improve patient outcomes. Securing the diagnosis by objective testing is the first step. Identifying the GERD phenotype is the second step. Engaging the patient to become aware of the symptoms’ mechanisms, the impact of the disease on quality of life, and the importance of adherence to healthy dietary and lifestyle measures is the third step. At this point, shared decision-making with the patient regarding therapeutic options is critical and should be based on long-term safety and on patient preferences to reduce decision regret [12^▪^]. Since GERD represents a spectrum of syndromes with distinct underlying pathophysiological characteristics, the choice of therapy depends on the clinical phenotype as defined by the predominant symptoms and the results of diagnostic investigations [13]. The response to PPI therapy, the severity and the circadian pattern of reflux, the deterioration of esophageal motility, the degree of esophageal mucosal changes, and the patient's psychological profile should be taken into account before deciding on surgery and the choice of the most appropriate procedure. The final goal is to tailor the surgical procedure to achieve a trade-off between reflux control and the risk of troublesome side-effects.

### Indications for surgical therapy and patient selection

All patients who are potential candidates for an antireflux procedure should undergo a thorough preoperative assessment to determine the anatomical and physiological characteristics of the foregut. Besides symptom evaluation, an appropriate work-up should include esophagogram, esophageal manometry, and ambulatory pH/impedance monitoring. In selected patients, especially those with ineffective peristalsis, gastric emptying scintigraphy should also be obtained. Candidacy for ARS includes confirmatory evidence of pathologic (acid) GERD, exclusion of achalasia, and assessment of esophageal peristaltic function [14]. Overall, well established predictors of success of laparoscopic antireflux surgery are the presence of typical symptoms, an abnormal 24-h pH score off PPI, and a good clinical response to acid suppression therapy with PPI [15].

### Basic technical aspects of antireflux surgery

Besides patient-related factors, technical factors predict success or failure of ARS. Optimal performance of ARS requires a clear understanding of GERD pathophysiology, structured training in laparoscopic and/or robotic surgery, and completion of an adequate learning curve for all technical components of the procedure. Dissection of the EGJ must be careful to avoid damage to the esophageal wall and to preserve the vagal nerves. An extensive lower mediastinal dissection is necessary to obtain a tension-free, intra-abdominal esophageal segment of 3–5 cm in length. In patients with medium to large size hiatal hernias, a posterior or composite crural repair including the anterior and left-lateral aspect of the hiatus is performed. Appreciation of the variable anatomical relationships between the hiatus, the EGJ, and the gastric fundus is important to construct a geometrically correct fundoplication [16,17]. Division of short gastric vessels and the gastrophrenic ligament, and removal of the fat pad at the angle of His is necessary to adequately mobilize the anterior and posterior gastric fundus. While wrapping the fundus around the distal esophagus, either posteriorly or anteriorly, the surgeon should make sure that the fundoplication is tension-free and symmetrical and that the fundus can loosely rotate clockwise or counter-clockwise with the pivot being the angle of His. Intraoperative measurement of the cross-sectional area and distensibility of the EGJ by endoluminal functional lumen imaging probe (EndoFLIP) technology may be useful to tailor the degree of crura repair and LES augmentation [18].

### Selection of the antireflux procedure

In general, ARS is safe, effective, and durable when performed in specialized centers. A multicenter European trial comparing medical therapy with fundoplication by expert surgeons showed that 92% of medical patients and 85% of surgical patients remained in remission at 5 years of follow-up [19]. However, despite remarkably low morbidity and mortality rates and excellent long-term outcomes [20], ARS is still underused due to the perceived risk of long-term side effects [21]. The inconsistency of reported clinical outcomes has restricted the adoption of fundoplication mainly to patients with long-lasting GERD and large hiatal hernia. An encircling wrap that is too tight, too long, or twisted due to tension from intact short gastric vessels, or a wrap made with the body rather than the fundus of the stomach can cause persistent dysphagia requiring revisional surgery in up to 9% of patients [22].

*Nissen fundoplication* has shown better efficacy in controlling reflux compared to partial fundoplication. However, recent guidelines suggest that the choice of ARS should be dictated on whether the patient prioritizes symptom improvement or minimizing postoperative side-effects [23]. The *posterior Toupet fundoplication* has emerged as a primary antireflux procedure or is offered to selected patients with nonspecific esophageal motility disorders to minimize the incidence of postoperative dysphagia [24]. Systematic reviews and randomized trials have shown that the Toupet fundoplication can decrease dysphagia, gas-bloating, and need of dilatation and reoperation, while providing similar reflux control compared to the Nissen fundoplication [25,26].

Use of the *anterior Dor fundoplication* can further minimize the risk of dysphagia since the intra-abdominal esophagus is not pushed forward or angulated by the posterior fundic wrap [27,28^▪^]. Watson *et al.* compared the outcomes of a modified (no division of short gastric vessels) 180° anterior fundoplication with the Nissen fundoplication and found no significant differences for reflux symptoms, side effects, and overall satisfaction at 10 years. The reoperation rate was lower with anterior fundoplication due to a lower incidence of dysphagia and hiatal hernia [29]. Supporting data for the anterior 90° and the anterior 120° partial fundoplication is less robust, with reflux being more common at 5-year follow-up [30–31]. A systematic review and meta-analysis of randomized control trials assessed the effectiveness of laparoscopic anterior (180° and 90°) versus laparoscopic Nissen (360°) fundoplication. The study included 4 eligible trials with 398 patients. At 5-years of follow-up, the risk of dysphagia for solids was higher after the Nissen procedure than after anterior fundoplication, while the risk of postoperative GERD medication use was lower with the Nissen. No differences were found in terms of heartburn, reoperation, and satisfaction rates [32].

*Magnetic sphincter augmentation (LINX procedure)*, especially in combination with crural repair, is highly effective in reducing GERD symptoms, PPI use, and esophageal acid exposure, and in improving quality of life [33,34]. The procedure is performed using the LINX device, a ring of magnets placed laparoscopically around the EGJ. Only limited surgical dissection with preservation of the phrenoesophageal ligament and no division of short gastric vessels is necessary in the absence of hiatal hernia [35]. Safety issues such as device erosion or migration have been rare and not associated with mortality [36]. The LINX device can be removed laparoscopically if necessary, thereby preserving the option of fundoplication or other therapies in the future. Contraindication to undergo scanning in high-power Tesla magnetic resonance systems remains a potential limitation. The LINX device was designed to limit the technical variability that occurs with fundoplication in patients with early progressive disease. Six- to 12-year outcome data in 124 patients implanted with Linx at a single institution and followed for a median of 9 years show that the mean GERD-HRQL score decreased from 19.9 to 4.01 at the latest office visit, the prevalence of grade 2–4 regurgitation decreased from 59.6% to 9.6%, and 79% of patients discontinued use of PPI. The mean percentage time pH <4 decreased from 9.7% to 4.2%. Four patients who had received radiofrequency ablation treatment for Barrett's esophagus without dysplasia before the Linx implant, and had esophageal acid exposure normalized after surgery, were followed for up to 8 years without recurrence of intestinal metaplasia. Predictors of a favorable outcome were age at intervention <40 years, and total GERD-HRQL score >15 [37]. Patients should have sufficient esophageal contractility or peristaltic body reserve to overcome the resistance imposed by the LINX device and its surrounding fibrous capsule. Although pneumatic dilation is effective in 67% of patients with persistent postoperative dysphagia, some patients require removal of the device. Preoperative identification of manometric abnormalities would be useful to stratify patients with an increased risk of persistent dysphagia. Adequate peristaltic reserve after multiple rapid swallows correlates with decreased incidence of dysphagia following LINX implantation [38,39]. In a multicenter study of 210 patients, 105 with ineffective esophageal motility (IEM) and 105 without IEM, independent risk factors for needing endoscopic dilation or device removal included age >45 years, preoperative dysphagia, MSA size <15 beads, and <40% intact swallows on preoperative manometry. All patients requiring removal had high resolution manometry (HRM) showing a distal contractile integral (DCI) <200 mmHg and <20% intact swallows [40^▪^]. Further prospective studies with high-quality pre and postoperative HRM data are definitely needed to understand the thresholds of baseline physiologic impairment that can still be effectively treated with LINX.

*Refluxstop* is a novel antireflux procedure designed to stabilize the EGJ in the abdomen using a small silicon device implanted laparoscopically in a pocket of the gastric fundus positioned above the level of the LES. The implant is preceded by the following steps: extensive esophageal dissection and cruroplasty, accentuation of the angle of His and restoration of the gastroesophageal flap valve, and creation of an anterior 90° fundoplication [41]. Division of the short gastric vessels and complete mobilization of the posterior aspect of the fundus are mandatory steps to perform an adequate fundoplication and to enable invagination of the prosthetic device in the gastric fundic pocket. The device provides stability to the EGJ by blocking the upward movement of the LES, prevents unfolding or mediastinal herniation of the fundoplication, and maintains the anatomically correct position of the angle of His. Early and medium-term results of this procedure confirm that it has the potential to permanently correct GERD. The recently reported 3-year results with this procedure in 50 patients treated by Refluxstop showed no serious adverse events related to the device. The average GERD-HRQL score decreased 93.1% from baseline. No significant dysphagia occurred, and daily regurgitation improved by 97.9% from baseline. The mean percentage total time with pH<4 decreased from 16.35% to 0.80% [42^▪^]. Interestingly, the Refluxstop procedure seems feasible, safe and effective even in GERD patients with IEM [43,44]. This procedure seems a reasonable trade-off between reflux control and risk of unwanted side-effects and should be tested in randomized clinical trials.

### Personalization of antireflux surgery for Barrett's patients

Barrett's patients with symptomatic GERD have historically been treated similar to GERD patients without Barrett's, with surgery being reserved for patients with persistent/refractory symptoms and late-stage disease. However, there is a need for precision, individualized treatment of Barrett's esophagus considering the risk of developing dysplasia and adenocarcinoma. The long-term results of ARS in patients with established Barrett's esophagus tend to be worse than in GERD patients without Barrett's esophagus. However, in early disease stages, surgery has the potential to reduce the likelihood of intestinal metaplasia developing within cardiac mucosa, to induce regression of intestinal metaplasia and low-grade dysplasia when present, and to reduce the overall risk of progression to esophageal adenocarcinoma [45,46]. In Barrett's patients, special attention should be paid to securely close the crura and address esophageal longitudinal tension to minimize the risk of recurrent GERD. Use of concurrent mucosal radiofrequency ablation (RFA) in patients with low-grade dysplasia may be a worthwhile adjunct to ARS. Long-term results from the SURF trial indicate that RFA results in sustained clearance of Barrett's mucosa and low-grade dysplasia in 91% and 96% of patients, respectively, after a median follow-up of 73 months [47].

### Personalization of antireflux surgery for patients with gastroesophageal reflux disease and obesity

In patients with proven GERD and morbid obesity (BMI >35), Roux-en-Y gastric bypass is an effective primary antireflux intervention [14] and provides additional benefits such as weight loss and improvement of comorbidities including diabetes, hypertension, and obstructive sleep apnea. Reflux control appears not inferior compared to fundoplication [48]. In individuals with BMI 30–35 and PPI-refractory GERD, primary standard ARS remains a valid option in expert hands [49^▪^]. Preoperative treatment with GLP-1 agonists and proper counselling can aid patients in meeting the BMI threshold for standard ARS rather than choosing Roux-en-Y gastric bypass [50].

### Personalization of antireflux surgery for patients with failed ARS

Studies on long-term failures of primary antireflux surgery show that re-herniation rates increase over time due to progressive deterioration and weakness of the central tendon and the left-lateral portion of the crura [51,52]. Whether the adjunct of hiatal mesh, Collis gastroplasty, relaxing diaphragmatic incisions, or anterior/posterior gastropexy may improve the outcomes of a standard crural repair with fundoplication and reduce hiatal hernia recurrence rates remains highly controversial [53,54]. Roux-en-Y gastric bypass is a salvage options for obese patients with BMI >35 presenting with recurrent GERD after primary ARS.

## CONCLUSION

Laparoscopic antireflux surgery for GERD and HH should be performed in referral centers providing comprehensive diagnosis and a spectrum of surgical techniques tailored to the individual patient and the GERD phenotype. The operation should be effective for reflux control, with limited side-effects, and durable. The choice of the surgical procedure should be based on shared decision-making with the patient considering disease severity and risk for disease progression. Targeting the individual components of EGJ competence may enable reflux control without total fundoplication and may decrease the incidence of side-effects.

## Acknowledgements

*None.*

### Financial support and sponsorship

*None.*

### Conflicts of interest

*There are no conflicts of interest.*

## REFERENCES AND RECOMMENDED READING

Papers of particular interest, published within the annual period of review, have been highlighted as:

▪ of special interest
▪▪ of outstanding interest
